# VoronaGasyCodes: A Public Database of Mitochondrial Barcodes for Malagasy Birds

**DOI:** 10.1111/1755-0998.70027

**Published:** 2025-08-07

**Authors:** Sushma Reddy, Kristen Wacker, Mai Fahmy, Evon Hekkala, John M. Bates, Steven M. Goodman, Shannon J. Hackett, Marie J. Raherilalao, J. Dylan Maddox

**Affiliations:** ^1^ Department of Fisheries, Wildlife, and Conservation Biology and Bell Museum of Natural History University of Minnesota St. Paul Minnesota USA; ^2^ Negaunee Integrative Research Center Field Museum of Natural History Chicago Illinois USA; ^3^ Department of Ecology and Evolutionary Biology and Museum of Zoology University of Michigan Ann Arbor Michigan USA; ^4^ Department of Biological Sciences Fordham University Bronx New York USA; ^5^ Association Vahatra Antananarivo Madagascar; ^6^ Mention Zoology and Animal Biology University of Antananarivo Antananarivo Madagascar

**Keywords:** DNA barcoding, iDNA, Madagascar, metabarcoding, mtDNA, species identification

## Abstract

Molecular tools are increasingly being used to survey the presence of biodiversity and their interactions within ecosystems. Indirect methods, like environmental DNA (eDNA) and invertebrate‐derived DNA (iDNA), are dependent on sequence databases with accurate and sufficient taxonomic representation. These methods are increasingly being used in regions and habitats where direct detection or observations can be difficult for a variety of reasons. Madagascar is a biodiversity hotspot with a high proportion of endemic species, many of which are threatened or endangered. Here we describe a new resource, VoronaGasyCodes, a curated database of newly published genetic sequences from Malagasy birds. Our database is currently populated with six mitochondrial genes or DNA barcodes for 142 species including 70% of the birds endemic to the island and will be periodically updated as new data become available. We demonstrate the utility of our database with an iDNA study of leech blood meals where we successfully identified 77% of the hosts to species. These types of resources for characterising biodiversity are critical for insights into species distribution, discovery of new taxa, novel ecological connections and advancing conservation and restoration measures.

## 1 Introduction

Madagascar is universally renowned as a global hotspot of biodiversity with an extraordinary range of unique and endemic species as well as higher taxa (Antonelli et al. 2022; Gautier and Lowry III 2022; Ralimanana et al. 2022). Its prolonged history of geographic isolation—first from Africa (≈130–118 million years ago) and then from India (≈82 million years ago)—in combination with its diverse topography, complex geology and climatic zones has given rise to the evolution of distinct lineages for most taxonomic groups (Yoder and Nowak 2006). For example, 79% of the 11,866 native vascular plant species found in Madagascar are endemic (Gautier and Lowry III 2022), as are 71% of its 178 native freshwater fish (Sparks and Stiassny 2022), nearly 100% of the currently described 365 native frogs (Glaw et al. 2022) and 100% of its 174 native nonvolant mammal species (Goodman and Soarimalala 2022). Discoveries of new species and range extensions are being made every year, as new surveys and modern tools are used to learn more about this unique biota (Antonelli et al. 2022).

The Malagasy avifauna is similarly characterised by high levels of endemism, where 52% of the island's 210 breeding bird species, along with two orders, four families and 40 genera are found nowhere else in the world (Safford et al. 2022). This level of endemism has drawn considerable interest from ornithologists, yet surprisingly fewer than half the endemic species have any DNA sequence data available on public repositories like GenBank (Reddy 2014; Šmíd 2022). The lack of genetic data can result in an underestimation of species diversity, which is especially problematic for a tropical area with high levels of biodiversity superimposed on intense conservation threats (Schmidt et al. 2023). Madagascar faces pressures to its unique biodiversity via overexploitation of biological resources and unsustainable agricultural practices (Ralimanana et al. 2022; Vieilledent et al. 2018). Yet, even as extinction rates become intensified, recent field surveys are leading to new species descriptions and improved understanding of Malagasy biodiversity (Antonelli et al. 2022; Gautier and Lowry III 2022; Ralimanana et al. 2022).

Molecular identification methods such as DNA barcoding, environmental DNA (eDNA) and invertebrate‐derived DNA (iDNA) are increasingly being applied to augment field survey methods (Creer et al. 2016). With the ease and cost‐effectiveness of next‐generation sequencing, these techniques have a variety of uses from species identification to revealing a range of community interactions. For example, eDNA samples from soils, freshwater systems, permafrost and even air can generate biodiversity inventories of these habitats (Ariza et al. 2023; Littlefair et al. 2022; Lynggaard et al. 2022; Monteath et al. 2023). Similarly, DNA barcoding and metabarcoding are extremely useful for unravelling trophic interactions, such as predator–prey dynamics (Riaz et al. 2023). DNA can also be obtained from the guts or surfaces of invertebrates (iDNA), serving as non‐invasive vertebrate surveys and revealing host–parasite interactions that are important for our understanding of both trophic interactions and disease dynamics (Fahmy et al. 2019, 2020, 2023; Schnell et al. 2012).

High‐quality reference databases of barcodes, derived from morphologically identified and archived museum specimens, are essential to the quality and success of metabarcoding studies. Given the urgent need for knowledge in this biodiversity hotspot, several databases have been created for specific taxonomic groups in Madagascar, such as reptiles (Nagy et al. 2012), amphibians (Perl et al. 2014), fishes (Vences et al. 2022), ants (Smith et al. 2005) and micromoths (Lopez‐Vaamonde et al. 2019). Furthermore, a few publicly accessible global databases have been developed, such as the Barcode of Life Database (BOLD; Ratnasingham and Hebert 2007). However, the completeness of such databases is a fundamental limitation to the identification of species in metabarcoding research (Keck et al. 2023). Indeed, a major motivator for this study was the difficulty we encountered in reliably identifying avian species in the blood meals of Malagasy leeches due to the lack of appropriate reference material from either the BOLD database or GenBank.

The need for first‐generation mtDNA sequence data may seem counterintuitive given the abundance of available bird genomes (Feng et al. 2020) and ongoing efforts to sequence the genomes of all extant bird species (Zhang 2015). Actively curated databases, however, are integral to the effectiveness of techniques employing mtDNA markers because the inferences from these studies depend heavily on the accuracy and quality of the sequence data and the accompanying metadata of specimens (Toczydlowski et al. 2021). Although GenBank is undoubtedly an unparalleled resource for genomic studies, its extensive scale and rapid growth have resulted in inaccuracies, as highlighted by recent research that has emphasised the prevalence of errors in model genomes (Steinegger and Salzberg 2020) and various taxa including fish (Li et al. 2018), mammals (Prada and Boore 2019) and amphibians (van den Burg et al. 2020). Birds have not escaped these errors, especially in mtDNA markers and may even occur at greater rates than other taxa (Sangster and Luksenburg 2021; van den Burg and Vieites 2023).

To address the lack of published genetic data on birds from Madagascar and to create a reference database of these species for traditional barcoding and ecological metabarcoding research, we have created VoronaGasyCodes (https://github.com/sreddyumn/VoronaGasyCodes), an actively curated, openly accessible and dynamic database featuring six commonly used mtDNA genes for birds. ‘Vorona’ is the Malagasy word for bird and ‘Gasy’ is the term in Madagascar's native language used for all that is related to the island and its people (Voarintsoa et al. 2019). This reference database offers researchers verified, high‐quality mtDNA markers from vouchered museum samples of Malagasy birds in addition to data available from Genbank. We have populated version 1.0 of the database with 142 bird species, comprising over half of the currently recognised bird species occurring in Madagascar and about 75% of the endemics. We aim to continue to build this resource as sequence data of new species become available. To demonstrate the utility of VoronaGasyCodes, we use it to identify bird iDNA from leech blood meals collected in Ranomafana National Park (RNP), Madagascar.

## 2 Methods

### 2.1 Sequencing

We obtained samples from vouchered specimens for 109 Malagasy bird species from the Field Museum of Natural History (FMNH) and the Université d'Antananarivo, Mention Zoologie and Biologie Animale (formerly Université d'Antananarivo, Département de Biologie Animale [UADBA]). We sampled 79 species endemic to Madagascar and an additional 15 endemic to the Malagasy region (including nearby western Indian Ocean islands). Our sampling included 43 families and in‐depth coverage within all major radiations, that is, 18 of the 21 currently recognised Vangidae species, all 11 Bernieridae species, 9 of 10 Couinae species and all 4 Philepittidae species. Additionally, our database includes two IUCN Red List species of diving ducks ( *Anas melleri* and *Aythya innotata* ) as well as eight species listed as Category I or II on the Convention on International Trade in Endangered Species of Wild Fauna and Flora (CITES). A complete list of species and voucher specimens is provided in the supplemental data and GitHub.

We extracted DNA from frozen tissues using Qiagen's DNeasy Blood and Tissue Kit following the manufacturer's instructions. Using published primers (Table 1) we amplified fragments of six mitochondrial genes: ribosomal genes 12S and 16S, cytochrome c oxidase subunit I (COI), cytochrome b (CYTB), NADH dehydrogenase 2 (ND2) and NADH dehydrogenase 3 (ND3). Reactions were performed in 25 μL with a final concentration of 1× PCR Buffer, 200 μM dNTPs, 1.5 mM MgCl2 and 1 unit of Taq polymerase. The thermal protocol consisted of an initial denaturing step at 95°C for 5 min, followed by 35 cycles of 95°C for 90 s, 55°C for 30 s and 72°C for 1 min, and terminated with a final extension step at 72°C for 10 min. PCR products were visualised via electrophoresis and successful reactions were purified using AMPure XP beads with a 1.8× ratio of beads to PCR product. We then prepared samples for Sanger sequencing using BigDye Terminator v. 3.1 Cycle Sequencing Kits (Applied Biosystems, USA) following the manufacturer's instructions and ran them on an ABI 3730 DNA analyser (Applied Biosystems, USA). The resulting sequences were trimmed, aligned and manually curated in Geneious Prime 2023.1.1 (https://www.geneious.com). To identify any obvious errors, we conducted a BLAST search against the National Center for Biotechnology Information's (NCBI) nucleotide database and visually inspected all pairwise similarity values.

**TABLE 1:** Primers used to amplify target mitochondrial genes.

Gene	Primers	Primer sequence	Citation
12S	H1478 L1091	5′‐GAGGGTGACGGGCGGTGTGT‐3′ 5′‐CAAACTGGGATTAGATACCCCACTAT‐3′	Kocher et al. (1989)
16S	16scpF 16scpR	5′‐CGAGGGCTTTACTGTCTCTT‐3′ 5′‐CCTATTGTCGATATGGACTCT‐3′	Weiskopf et al. (2018)
CYTB	H15149 L14851	5′‐GCCCCTCAGAATGATATTTGTCCTCA‐3′ 5′‐CCTACCTAGGATCATTCGCCCT −3′	Kocher et al. (1989)
COI	BirdF1 BirdR1	5′‐TTCTCCAACCACAAAGACATTGGCAC‐3′ 5′‐ACTTCTGGGTGGCCAAAGAATCAGAA‐3′	Dove et al. (2008); Kerr et al. (2007)
ND2	H6313 L5758	5′‐CTCTTATTTAAGGCTTTGAAGGC‐3′ 5′‐GGNGGNTGAATRGGNYTNAAYCARAC‐3′	Johnson and Sorenson (1998)
ND3	H11151 L10755	5′‐GATTTGTTGAGCCGAAATCAAC‐3′ 5′‐GACTTCCAATCTTTAAAATCTGG‐3′	Chesser (1999)

### 2.2 Compiling Existing Data

We downloaded all relevant nucleotide data from GenBank using the search terms ‘Madagascar’ AND ‘birds OR Aves’ AND [gene name, as listed in Table 1]. We then double‐checked to only keep data from species found in Madagascar and matched them to our newly generated sequences. We also double‐checked the accuracy of species and locus identifications by conducting visual inspections of alignments and neighbour‐joining trees to ensure that sequences grouped with similar taxa based on known phylogenetic relationships. Including previously published data allowed us to begin to document intraspecific variation into our database since our strategy for generating new sequences was to aim for at least a single individual of each species.

### 2.3 Leech Sampling and Host Identification

We collected 530 terrestrial leeches (primarily *Chtonobdella fallax*, Family Haemadipsidae) in Ranomafana National Park (RNP), located in southeastern Madagascar (47°18′–47°37′ E and 21°02′–21°25′ S). Leeches were collected along transects perpendicular to designated trails to minimise human DNA contamination within blood meals. Leeches were used to survey vertebrate biodiversity in RNP, and comprehensive results across taxonomic groups can be found in Fahmy et al. (2019, 2020).

We dissected all leeches for a portion of the gastric caeca containing the blood meal. This tissue from each leech was individually processed for DNA extraction with Qiagen's DNeasy Blood and Tissue kit following the manufacturer's protocols. Prior to amplification and sequencing, we pooled 2 μL of each DNA extract into samples that reflected the same leech species and collection locality (by transect). We performed all subsequent amplification and sequencing on these pooled samples. We targeted mitochondrial genes 12S, 16S, COI and ND2 following the methods of Fahmy et al. (2020). Briefly, we amplified each locus twice to account for varied amplification success, once with the forward Illumina adapter (ACACTCTTTCCCTACACGACGCTCTTCCGATCT) 5′ to the forward primer and once with the reverse Illumina adapter (GACTGGAGTTCAGACGTGTGCTCTTCCGATCT) 5′ to the reverse primer. This approach helps mitigate potential read‐quality biases associated with R1 and R2 in paired‐end sequencing. Thermocycling conditions were as follows: an initial denaturation at 94°C for 1 min, followed by 40 cycles of 94°C for 15 s, annealing at 54°C (or 50°C for ND2 and COI) for 30 s and extension at 70°C for 45 s, with a final extension at 72°C for 2 min. We combined duplicate amplicons by pooled sample, cleaned with a 2:1 carboxylated bead‐to‐amplicon ratio and submitted for paired‐end 250‐bp sequencing on an Illumina MiSeq at GENEWIZ Inc. (Plainfield, New Jersey).

We trimmed resulting reads using Trimmomatic (v.0.38; Bolger et al. 2014) to remove primers and fragments under 100 bp. Next, we assembled reads by clustering at 98% similarity with USEARCH v.5 (Edgar 2010). We filtered these clusters to remove non‐vertebrate DNA with an initial BLAST against a local database containing whole vertebrate genomes. We queried remaining clusters against NCBI's nt database on GenBank. Our iDNA survey of leech blood meals recovered 5741 sequences that were identified as avian.

### 2.4 Identifying to Avian Hosts

We used the VoronaGasyCodes database to identify iDNA sequences to species by conducting a local blast using the rBLAST: R Interface for the Basic Local Alignment Search Tool v0.99.4 (Hahsler and Anurag 2024; doi: 10.18129/B9.bioc.rBLAST) R package and limited search results to ≥ 97% pair‐wise identity and ≥ 80 bp coverage across matches. For results matching multiple hits, we chose the match with the highest percent identity, ensuring it was also the hit with the least number of mismatches and highest coverage. We provide sample R scripts and Python codes to run local blast on the VoronaGasyCodes GitHub site.

## 3 Results and Discussion

Our sampling and sequencing generated a database containing 1740 sequences from 142 species that encompasses 72% (79/110) of Madagascar's endemic bird species. Sequencing success for the target genes ranged from 88% for COI to 94% for 12S and ND2. For all sampled species except one, we had no more than two genes that failed to amplify. We were only able to sequence two genes for one problematic sample of *Tyto soumagnei* . In addition, we extracted all available sequences of Malagasy birds from Genbank and included them in our database, resulting in a total of 142 species currently represented, including five extinct species. VoronaGasyCodes 1.0 includes the following sequences of each gene: 12S—156 (102 newly generated, 54 from GenBank); 16S—110 (94, 16); CYTB—479 (92, 387); COI—132 (93, 39); ND2—368 (100, 268); ND3—495 (101, 394).

To facilitate the dissemination of these data, we made the VoronaGasyCodes database available as supplemental information on GitHub (https://github.com/sreddyumn/VoronaGasyCodes) and also on Zenodo (DOI: 10.5281/zenodo.15825891). In addition to sequences, this site provides basic metadata: GPS locations, accession numbers and links to specimens when available. Newly sequenced data are also available on GenBank; however, we believe that the curated database on GitHub will be more accurate and user‐friendly for ecological and conservation‐oriented studies needing to compare genetic data across all Malagasy birds. GenBank does not provide the ability for public comments or corrections for individual sequences, and known errors are still present (van den Burg and Vieites 2023). Although it is impossible to prevent all errors, our database will provide a means to fix them efficiently, as GitHub provides the ability to flag problematic records and request changes. Thus, our database will be dynamic in terms of updates with new sequences and user input. Finally, using a local database will be far less computationally intensive than blasting the entire GenBank database. We also suggest following best practices for conducting metabarcoding studies with more than one gene (Axtner et al. 2019; Deiner et al. 2017; Fahmy et al. 2020) because some species are difficult, if not impossible, to identify without multiple genetic markers (see below).

**FIGURE 1:**
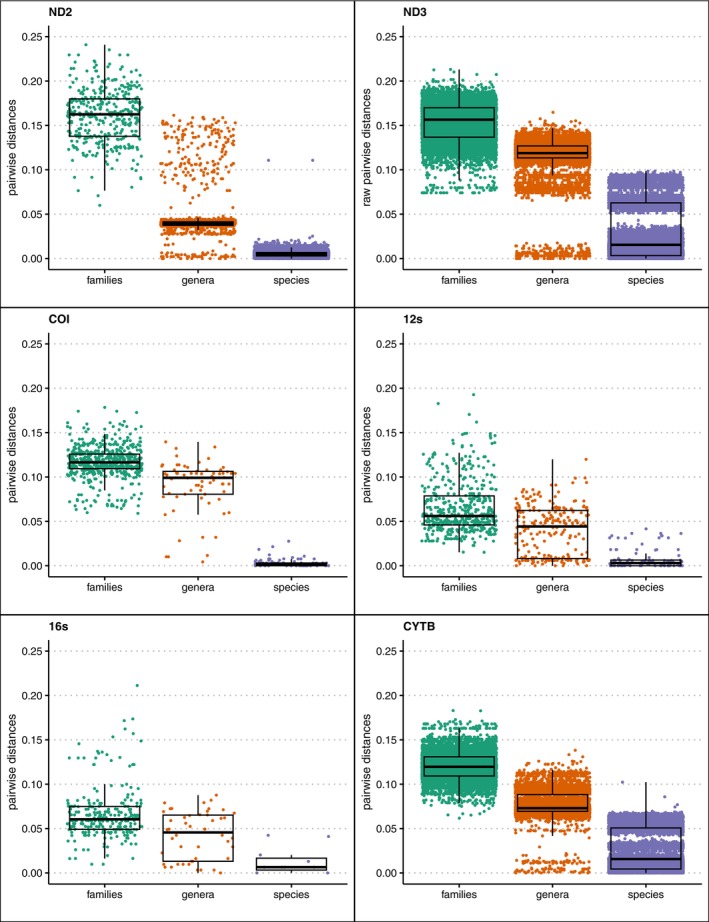
Box and whiskers plot showing uncorrected pairwise distances within species (purple), genera (orange), families (green) for each gene. Boxes indicate 25%–75% interquartile range with the dark lines showing median values. While all genes show the expected trends of median distances within species being less than within genera and these in turn being less than within families, in some cases distances between congeners were similar to within species. This likely indicates the need for taxonomic review of these taxa. Please note that the number of points varies in each plot due to the differences in the number of sequences available for each gene.

The potential utility of a barcoding gene is dependent on whether the locus can uniquely identify a species, which is dependent on genetic divergences being related to evolutionary distance. The variation of genetic distances differed across genes (Figure 1) and, apart from a few exceptions, were unique to species. Although all met the expectations of intraspecific distances being less than intrageneric, and those in turn being less than intrafamilial, there were some species that showed similar distances between congeners as they did within species (Figure 1). This is not an error of sequencing but rather the reality of genetic divergences (or lack thereof) in these species and points to the need for more systematic efforts to understand species limits in these groups (see Block et al. (2015) for a specific example of the potential infraspecific complexities known for Malagasy birds). Identical sequences between congeners were found in *Coua*, *Foudia*, *Monticola*, *Apus* and *Aepyornis*. Average pairwise genetic distances, calculated as the raw number of differences between two sequences, are shown in Table 2 and Figure 1 so users can make informed decisions about what genes to use in their study.

**TABLE 2:** Minimum, maximum, mean and standard deviation of pairwise genetic distances within species, within genera and within families for the six genes.

		Taxonomic rank	Minimum	Maximum	Mean	Standard deviation
12 s	*n* = 100	Species	0.000	0.042	0.008	0.011
	*n* = 218	Genus	0.002	0.120	0.041	0.027
	*n* = 458	Family	0.015	0.193	0.066	0.030
16 s	*n* = 11	Species	0.000	0.042	0.013	0.015
	*n* = 50	Genus	0.000	0.088	0.041	0.027
	*n* = 256	Family	0.010	0.211	0.066	0.029
COI	*n* = 97	Species	0.000	0.028	0.003	0.004
	*n* = 70	Genus	0.000	0.140	0.089	0.031
	*n* = 555	Family	0.059	0.178	0.117	0.018
CYTB	*n* = 12,133	Species	0.000	0.102	0.027	0.024
	*n* = 8321	Genus	0.000	0.138	0.077	0.015
	*n* = 6404	Family	0.062	0.183	0.120	0.015
ND2	*n* = 6408	Species	0.000	0.111	0.005	0.004
	*n* = 1960	Genus	0.000	0.161	0.045	0.026
	*n* = 390	Family	0.060	0.264	0.165	0.036
ND3	*n* = 12,336	Species	0.000	0.099	0.030	0.031
	*n* = 8330	Genus	0.000	0.165	0.116	0.022
	*n* = 10,416	Family	0.074	0.213	0.153	0.021

**FIGURE 2:**
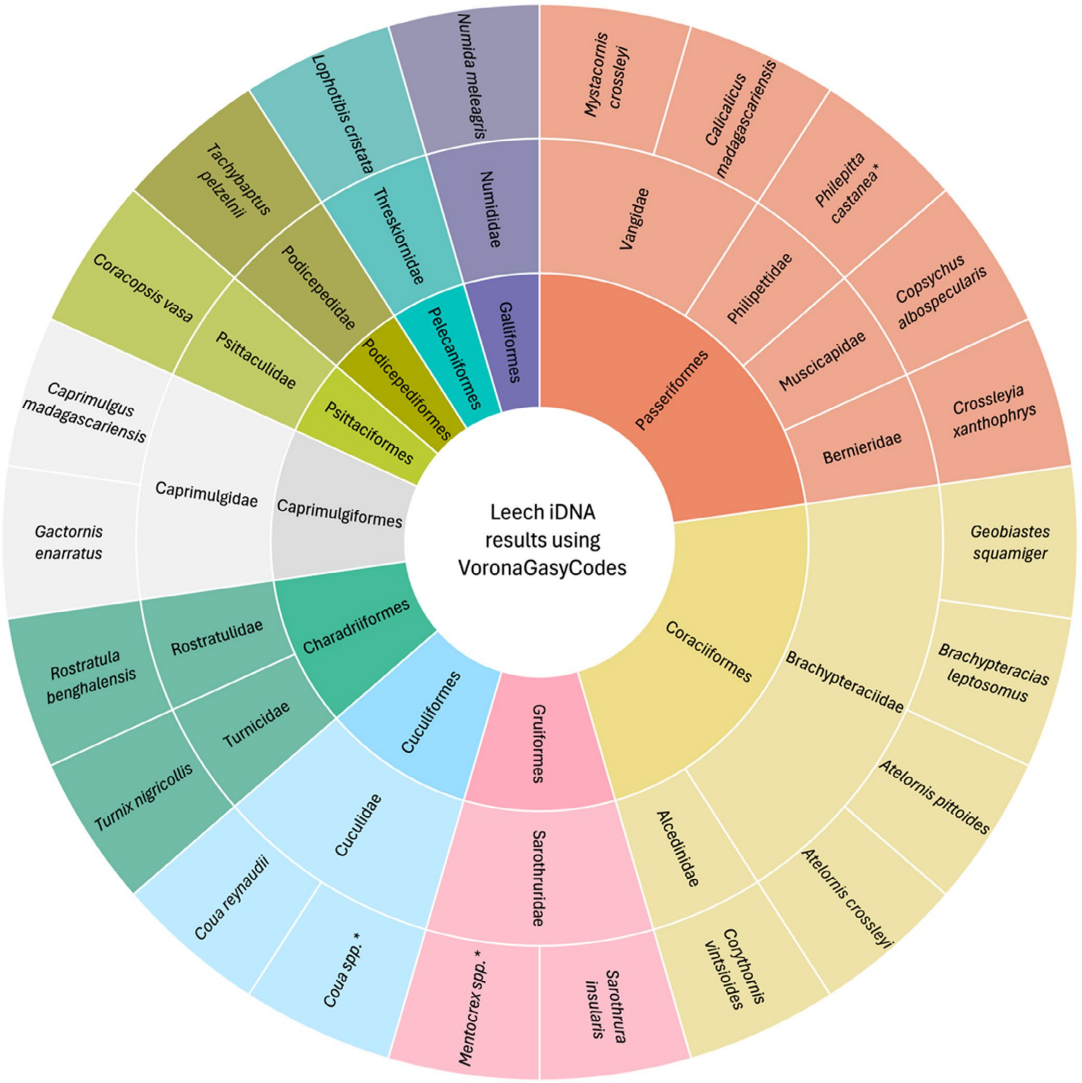
Taxonomic diversity of leech bloodmeal matches after blasting to VoronaGasyCodes. Each colour represents a different order and concentric circles going from inside to out indicate orders, families and species detected by this study. * = uncertain species assignments.

We successfully identified 23 different bird species from 530 leeches collected and analysed (Table 3; Figure 2). Of the 5741 sequences, we matched 4351 sequences at 97% or higher. The 5741 sequences were 2183 unique strings, of which 1539 matched to a bird species in our sequences and 644, which did not match, are likely from species not in our database. Note that these numbers do not translate to the number of species because the iDNA sequences are short (< 250 bp) and may be from different, non‐overlapping parts of larger gene regions.

**TABLE 3:** Local BLAST matches of leech blood meal iDNA compared to VoronaGasyCodes sequences.

Genus	Species	ND2	COI	16S	12S
*Atelornis*	*crossleyi*				15
*Atelornis*	*pittoides*	277		271	280
*Brachypteracias*	*leptosomus*				8
*Calicalicus*	*madagascariensis*			2	
*Gactornis*	*enarratus*				99
*Caprimulgus*	*madagascariensis*				12
*Copsychus*	*albospecularis*	8	38	349	369
*Coracopsis*	*vasa*				2
*Corythornis*	*vintsioides*				5
*Coua*	*reynaudii*	3	84	383	781
*Coua*	spp.^a^			2	15
*Crossleyia*	*xanthophrys*			2	
*Geobiastes*	*squamiger*				8
*Lophotibis*	*cristata*	9	7	676	381
*Mentocrex*	spp.^a^				15
*Mystacornis*	*crossleyi*				1
*Numida*	*meleagris*				2
*Philepitta*	*castanea* ^a^				94
*Rostratula*	*benghalensis*				4
*Sarothrura*	*insularis*				105
*Tachybaptus*	*pelzelnii*				42
*Turnix*	*nigricollis*			2	
	Total	297	129	1687	2238

^a^ Indicate taxon identifications with some uncertainty (see text for more details).

While we have confidence in most of our matches based on high percent identity and knowledge that these bird species are known to occur in RNP, in a few cases we were uncertain about the blast results (noted with * in Table 3). This included three cases of matches near 97% percent identity that warranted more scrutiny. First, there were 15 matches in 12S and 2 matches in 16S to couas that were somewhat suspicious given that these sequences matched equally high to several *Coua* species. We report these matches as *Coua* spp. until we can gather more data to resolve to species. Next, there were 15 matches to *Mentocrex beankaensis* in 12S; however, this species does not occur in RNP but rather its sister species *M. kioloides* does. We did not have *M. kioloides* in our database; therefore, the closest hit from the VoronaGasyCodes sequences was to *M. beankaensis*. To be most accurate, we report these matches as *Mentocrex* spp. Finally, we discovered matches to *Philepitta castanea* and *Philepitta schlegeli* in 12S were equally likely, meaning they had similar percent identity, mismatches and coverage. Given that *P. schlegeli* does not occur in RNP, it is most likely that these all should be identified as *P. castanea* .

The potential to match a DNA sequence is dependent on several factors in addition to whether there is enough representation in the database. Given the nature of DNA sequencing technologies, the data generated by next‐generation sequencing (as we did for the leech‐derived DNA) is often short (< 200 basepairs [bp]) while Sanger sequencing (which we used for the bird genes) allows for longer reads (up to ~800 bp). The genes targeted in VoronaGasyCodes ranged from 300 to 500 bp and in some cases the data available on Genbank were longer fragments. The overlap of the query and reference sequences also contributes to the confidence in matches. In most cases, using stringent settings in blast queries can overcome this issue. However, as discussed above, it may be hard to distinguish congeneric species if the coverage overlap is small. If there are multiple hits with high percent identity, manual curation is necessary to examine the extent of coverage and if the match is reasonable.

The matching bird species from our iDNA survey are known to occur in the sampled region and exhibit a range of body sizes, trophic levels, feeding ecologies and nesting behaviours. These species are variously classified as terrestrial, arboreal, generalist and aquatic in their lifestyles (Razafindratsima et al. 2018; Tobias et al. 2022), showing that surveys through leech sampling can capture a range of species with different ecologies within the sampled area. The four most common birds found in the blood meals were *Coua reynaudii* (Red Fronted Coua), *Lophotibis cristata* (Madagascar Ibis), *Copsychus albospecularis* (Madagascar Magpie‐Robin) and *Atelornis pittoides* (Pitta‐like Ground‐Roller), all of which are generalist birds that forage on or near to the ground, in trees, or stream edges (Kirwan et al. 2020; Langrand and Kirwan 2020; Matheu et al. 2020; Payne 2020) and are known to occur in RNP.

Apart from understanding the feeding dynamics of Malagasy leeches, identifying hosts from blood meals also provides a unique opportunity to survey birds from different microhabitats. For instance, previous studies show that leech gut sampling can include DNA of up to four hosts (Fahmy et al. 2020). Leeches exhibit geographic site fidelity (Tessler et al. 2018) and have low dispersal rates but are generally easier to sample than birds. Furthermore, leech blood meals can retain host DNA in their guts on the order of months, making them a useful tool for biodiversity assessments at sites where host species are difficult to monitor (Fahmy et al. 2019).

Our database also brings to light the possibility of cryptic species, taxa that are genetically but not always morphologically distinct and therefore often unrecognised in current classifications. The avian species diversity of Madagascar is considered relatively low for the size of the island (Schulenberg 2003), but recent molecular studies have identified a number of cryptic species as well as evidence of microendemism (e.g., Goodman et al. 1996; Younger et al. 2018, 2019), suggesting the true number of bird species may be higher. The VoronaGasyCodes database could serve as an initial screen for cryptic species, where researchers using iDNA or eDNA can identify hits with high confidence (known species) and those that do not match (potential new or rare species not represented in our database). This functionality will become more useful in the future as we continue to populate the database with more sequences from different locations and previously unrepresented species.

In conclusion, VoronaGasyCodes is an actively curated database of commonly used mitochondrial genes that provides researchers with verified and high‐quality markers for species identification. We have demonstrated its potential utility through a study of iDNA samples from Madagascar that enabled the identification of host bird species. Our database covers a majority of the bird species found in Madagascar. We included all the Malagasy birds for which we could obtain tissue samples; however, there are still many species not represented in modern genetic resources collections. We aim to continuously update the VoronaGasyCodes database and release new versions of the database as new data are generated for Malagasy birds. We invite researchers working in Madagascar to contribute to the database by notifying us about any potential problems and submitting data as they become available. We aim to address any problematic samples or new submissions whenever possible within a month of receipt. We will also aim to release new versions of the database each year unless no new data are produced. By collaborating, sharing and disseminating data, we can further enhance the accuracy and usefulness of this valuable tool to facilitate future studies of the distinctive Malagasy avifauna.

## Author Contributions

S.R.M.F., E.H., J.M.B., S.M.G., S.J.H., M.J.R., K.W. and J.D.M.: designed the study. M.F., E.H., J.M.B., S.M.G., S.J.H. and M.J.R.: contributed to sample acquisition. S.R., K.W., M.F., E.H. and J.D.M.: performed the research. S.R., K.W., M.F. and J.D.M.: analysed the data. S.R., K.W. and J.D.M.: wrote the original draft. All authors reviewed and edited the manuscript.

## Disclosure

*Benefit‐Sharing*: This study is the result of a research collaboration with scientists from Madagascar and the United States. Collaborators shared genetic samples and helped to design the study. All contributors are included as co‐authors. We are committed to international scientific partnerships and institutional capacity building. This research and the resulting database and publication are freely accessible to everyone, a key benefit we hope will empower Malagasy scientists who are at the forefront of conservation efforts in the biodiversity hotspot.

## Conflicts of Interest

The authors declare no conflicts of interest.

## Supporting information


**Data S1:** men70027‐sup‐0001‐Supinfo.zip.

## Data Availability

All bird genetic data are available as fasta files in supplemental information, the VoronaGasyCodes site at https://github.com/sreddyumn/VoronaGasyCodes, Zenodo (DOI: 10.5281/zenodo.15825891) and on Genbank (newly generated data have accession nos. PV916129‐PV916230 [12S]; PV911314‐PV911407 [16S]; PV915331‐PV915423 [COI]; PV932719‐PV932810 [CYTB]; PX068536‐PX068635 [ND2]; PV995247‐PV995347 [ND3]). All specimen metadata can be found on the VoronaGasyCodes GitHub site, on Zenodo and as Supporting Information S1. All leech‐derived sequences are available on GenBank, BioSample accession no. SAMN49765806.
